# Beyond the vision: central serous chorioretinopathy, anxiety and depression–a systematic review

**DOI:** 10.3389/fmed.2025.1758428

**Published:** 2026-01-12

**Authors:** Alessandro Meduri, Emanuele Maria Merlo, Giorgio Sparacino, Maura Mancini, Giovanni William Oliverio, Orlando Silvestro, Laura De Luca, Gabriella Martino, Pasquale Aragona

**Affiliations:** 1 Ophthalmology Clinic, Department of Biomedical and Dental Sciences and Morphofunctional Imaging, University of Messina, Messina, Italy; 2 Department of Biomedical and Dental Sciences and Morphofunctional Imaging, University of Messina, Messina, Italy; 3 Course Degree in Medicine and Surgery, University of Messina, Messina, Italy; 4 Department of Health Sciences, University Magna Graecia of Catanzaro, Catanzaro, Italy; 5 Department of Clinical and Experimental Medicine, University of Messina, Messina, Italy

**Keywords:** anxiety, central serous chorioretinopathy, clinical psychology, depression, maculopathy, ophthalmology, psychopathology

## Abstract

**Background:**

Central Serous Chorioretinopathy (CSCR) is an exudative maculopathy characterized by serous retinal detachment, choroidal hyperpermeability, and retinal pigment epithelium dysfunction. Beyond its ocular features, CSCR has long been associated with stress-related mechanisms and emotional dysregulation. Psychological factors such as anxiety and depression may influence disease onset, recurrence, and recovery; however, no previous review has systematically synthesized quantitative evidence on these variables in CSCR.

**Methods:**

This systematic review followed the PRISMA 2020 guidelines and was registered in PROSPERO (CRD420251160719). PubMed, Scopus, and Web of Science were searched in October 2025 using the terms *“Central Serous Retinopathy” AND Anxiety AND Depression*. Eligible studies included peer-reviewed, English-language articles employing standardized psychometric instruments to assess anxiety and/or depression in CSCR patients. Twenty studies met the inclusion criteria, comprising 19 case–control and one longitudinal cohort design.

**Results:**

Across the included studies, depressive symptoms were significantly higher in CSCR patients in most investigations, indicating a consistent affective vulnerability. Findings for state anxiety were more heterogeneous, with approximately half of the studies reporting significant group differences. Evidence on trait anxiety was limited but suggested potential links with physiological dysregulation, including altered neurovascular coupling. Overall, psychological distress-particularly depressive symptoms-emerged as a frequent and clinically meaningful correlate of CSCR.

**Conclusion:**

The present review provides the first comprehensive synthesis of quantitative evidence linking anxiety and depression with CSCR. These findings highlight the need for routine psychological screening and integrative management in ophthalmology. Future longitudinal and interventional research is warranted to clarify causal pathways and evaluate targeted psychological or stress-reduction interventions, ultimately promoting holistic care that addresses both the visual and emotional dimensions of this condition.

**Systematic review registration:**

https://www.crd.york.ac.uk/PROSPERO/view/CRD420251160719

## 1 Introduction

Central serous chorioretinopathy (CSCR) is a common exudative maculopathy characterized by serous retinal detachment, typically involving the macula, and often associated with pigment epithelial detachment, retinal pigment epithelial (RPE) dysfunction, and choroidal thickening with venous overload and hyperpermeability (1–4). It represents the fourth most frequent non-surgical retinopathy (5, 6) and is generally classified as acute, chronic, or recurrent, depending on disease duration, and as simple or complex, according to the extent of RPE changes visible on fundus autofluorescence. Recent epidemiological projections indicate that approximately 1.97 million individuals will develop CSCR in 2025, rising to 2.03 million in 2030, 2.30 million in 2040 and 2.43 million in 2050 (1).

CSCR frequently coexists with other ocular and systemic disorders. The most prevalent macular comorbidities include non-proliferative diabetic retinopathy, non-exudative age-related macular degeneration, and hypertensive retinopathy, whereas common non-macular conditions comprise lattice degeneration, optic atrophy, rhegmatogenous retinal detachment, and optic disk pit (7). Among these, diabetes is the most frequent and clinically significant comorbidity, representing a major source of systemic stress and ophthalmic complications (8–12).

Beyond these biological aspects, CSCR has long been regarded as a condition strongly linked to psychophysiological stress and maladaptive emotional regulation. Several studies suggest that chronic stress exposure may lead to cortisol dysregulation, endothelial dysfunction, and choroidal vascular hyperpermeability, mechanisms potentially contributing to disease onset and recurrence (13–16). Moreover, trait-related psychological vulnerability-including anxiety sensitivity, depressive reactivity, and difficulties in emotional processing- may modulate both autonomic imbalance and adherence to medical treatments, thereby influencing visual prognosis and recovery (17–19). Such evidence underscores the need to integrate psychological and physiological perspectives in understanding CSCR as a multifactorial disorder in which emotional distress, stress hormones, and vascular mechanisms jointly shape the clinical course.

In recent years, an increasing body of literature has highlighted the relevance of psychological factors, including anxiety, depression, stress reactivity, and coping, in determining the course of medical conditions (17–24). These factors play a key role in modulating adjustment to illness, treatment adherence, and clinical outcomes across a wide range of chronic diseases (25–30). Despite such evidence, certain ophthalmological conditions - such as CSCR - remain underexplored from a psychosomatic perspective.

Notably, Scott et al. (28), through a systematic review and meta-analysis, emphasized that anxiety and depression not only predict the onset of several chronic conditions but also affect disease adjustment and treatment adherence. Increased anxiety and depressive symptoms are associated with unfavorable clinical trajectories, poorer quality of life, and higher relapse risk. While previous reviews have investigated general psychopathology and personality traits in CSCR (31, 32, 81), to the best of our knowledge, no systematic review has previously synthesized quantitative evidence on anxiety and depression in patients with CSCR, including the most recent contributions.

Accordingly, the present systematic review aims to provide the first comprehensive synthesis of studies assessing anxiety and depression in patients with CSCR. Understanding the interaction between emotional distress and retinal pathology may contribute to improved patient care and inform the design of tailored psychological and interdisciplinary interventions, fostering better adjustment to this complex visual condition.

## 2 Methods

This systematic review was conducted in accordance with the Preferred Reporting Items for Systematic Reviews and Meta-Analyses (PRISMA) guidelines to ensure methodological rigor and transparency (33–35). The PRISMA 2020 Checklist is provided in the Supplementary materials. The review protocol was registered in PROSPERO (CRD420251160719).

### 2.1 Search strategy and information sources

A systematic search was performed in PubMed, Scopus, and Web of Science between 1 July 2025 and 1 November 2025 to identify relevant studies. The search string used was *“Central Serous Retinopathy” AND Anxiety AND Depression* (see Table 1 for the complete list of terms). Only original research articles published in English and providing novel quantitative data were considered eligible. No time restrictions were applied.

**Table 1:** List of search terms.

Number	Terms
1	Central serous retinopathy
2	Anxiety*
3	Anxiety
4	Anxiety disorder
5	Depress*
6	Depression
7	Depressive disorder
8	Depressive SYMPTOMS
9	2 OR 3 OR 4
10	5 OR 6 OR 7 OR 8
11	1 AND 9 AND 10

Search terms were grouped by domain: ophthalmologic (row 1), psychological (rows 2–10), and combined (row 11).

### 2.2 Inclusion and exclusion criteria

Studies were considered eligible if they met the following conditions: they were full-text, peer-reviewed articles published in English, involving participants with a confirmed diagnosis of Central Serous Chorioretinopathy (CSCR). To ensure methodological robustness, only investigations that employed standardized psychometric instruments for the assessment of anxiety and/or depressive symptoms and provided quantitative data establishing a direct association between CSCR and psychological outcomes were included. In addition, previously published systematic reviews and meta-analyses were screened to identify potentially missing primary studies meeting these criteria.

Conversely, studies were excluded if they consisted of conference abstracts, qualitative research, narrative or systematic reviews, or case reports, as well as those relying on self-reported diagnoses of CSCR. Articles were also excluded when the investigated samples included participants with other chronic or mixed ophthalmological conditions, since such heterogeneity would have hindered a specific evaluation of the relationship between CSCR and psychological variables.

### 2.3 Selection and data collection

Two independent reviewers screened all retrieved records. Duplicates were removed, and titles and abstracts were examined to identify studies meeting the inclusion criteria. Full texts were then assessed for eligibility, and the reference lists of included papers were manually searched for additional relevant works. Data were extracted on authors, year of publication, country, study design, sample characteristics, assessment instruments, and main findings. Evidence was synthesized according to the domains of depression and anxiety (state and trait).

### 2.4 Quality assessment

The methodological quality of each included study was independently evaluated by two reviewers using the NIH Study Quality Assessment Tools. Disagreements were resolved through discussion until full consensus was achieved.

## 3 Results

**Figure 1:**
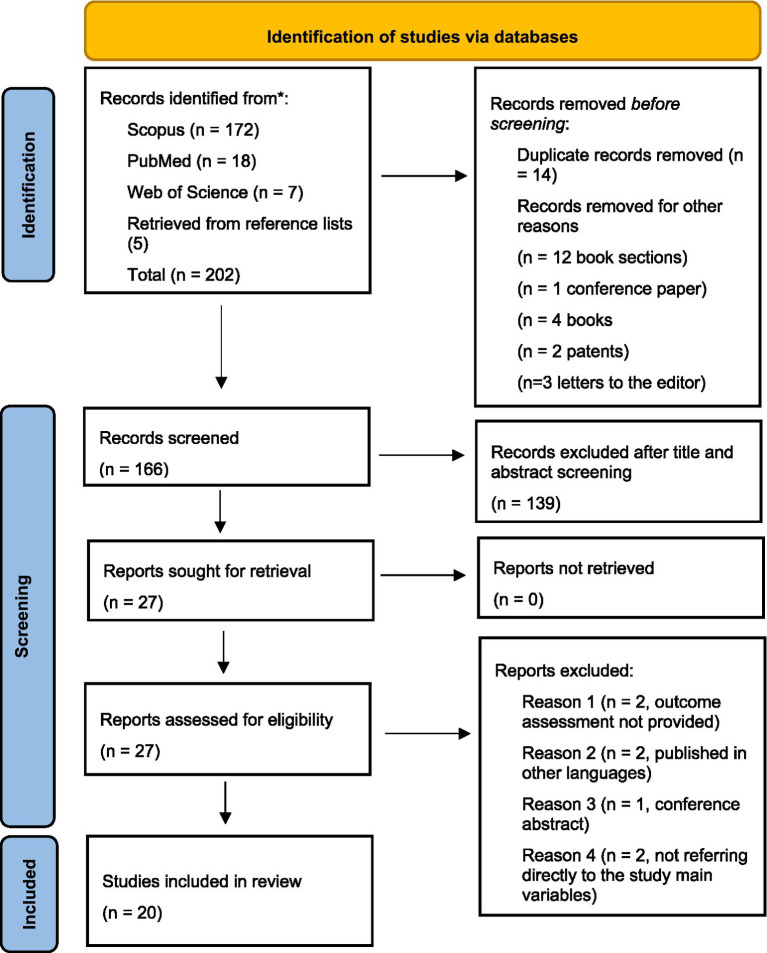
PRISMA flow diagram.

The results of the search and screening process are summarized in Figure 1, which illustrates the inclusion flow according to PRISMA guidelines.

A total of 202 records were initially identified as potentially eligible. After the removal of 14 duplicates and 22 records that did not meet the inclusion criteria, 166 studies remained for title and abstract screening. Of these, 139 were excluded as irrelevant or not aligned with the study objectives. The full texts of 27 articles were subsequently assessed for eligibility, and 7 were excluded because the outcome measures were not reported, the papers were published in languages other than English, or they consisted of conference abstracts or studies not directly addressing the target variables. Ultimately, 20 studies met all the inclusion criteria and were included in the final synthesis. Extracted data are summarized in Table 2.

**Table 2:** Main characteristics of the included studies.

Authors	Year	Study Design	Country	Sample	Assessment instruments	Main findings
Balkarli et al. (37)	2018	Case–control study	Turkey	58 CSCR patients 99 age-and-sex-matched healthy controls	Beck Anxiety Inventory (BAI) Beck Depression Inventory (BDI)	CSCR patients reported higher rates of sexual dysfunction than healthy controls, with scores inversely correlated with age and BMI. The BAI and BDI were used to assess anxiety and depressive symptoms in relation to sexual functioning.
Balkarli et al. (36)	2017	Case–control study	Turkey	83 CSCR patients 201 age-and-sex-matched healthy controls	Beck Anxiety Inventory (BAI) Beck Depression Inventory (BDI)	Significant differences in anxiety and depression were observed between patients diagnosed with both CSCR and fibromyalgia and those without CSCR (anxiety: p < 0.001; depression: *p* < 0.001), with higher scores in the CSCR + fibromyalgia group (anxiety: 16.83 ± 13.09 vs. 4.95 ± 6.93; depression: 14.34 ± 9.09 vs. 6.80 ± 6.96). However, neither anxiety nor depression emerged as predictive factors.
Bazzazi et al. (55)	2015	Case–control study	Iran Switzerland	30 CSCR patients 30 healthy age-and sex-matched controls	Hamilton Anxiety Rating Scale (HAM-A)	Anxiety levels were significantly higher in CSCR patients than in healthy controls (*p* < 0.001). No significant sex differences were observed. Regardless of whether the episode was a first or a recurrent CSCR presentation, patients consistently reported higher anxiety scores than controls.
Ben-Eli et al. (38)	2025	Case–control study	Israel	32 CSCR patients 8 DR patients 29 controls	Beck Anxiety Inventory (BAI)	CSCR patients tended to report lower life satisfaction and higher anxiety levels than healthy controls; however, these differences were not statistically significant (*p* = 0.19). Anxiety scores showed no significant correlations with pupil size, anterior chamber depth, amplitude of accommodation, or intraocular pressure.
Çam et al. (39)	2024	Case–control study	Turkey	55 CSCR patients 55 age-and-sex-matched healthy controls	Symptom Checklist 90-R (SCL-90R)	CSCR chronic patients presented significantly higher depression (*p* = <0.001) and anxiety (*p* = 0.002) rates than controls. Patients with acute CSCR were also significantly more depressed than controls (*p* = 0.007), although no significant differences were found for anxiety.
Conrad et al. (41)	2014	Case–control study	Germany	57 CSCR patients 57 age-and-sex-matched healthy controls	Symptom Checklist 90-R (SCL-90R)	CSCR patients showed significantly higher levels of emotional distress, anxiety, and depression than controls, scoring significantly higher on nine of the SCL-90-R subscales (i.e., anxiety: p < 0.001; depression: p < 0.001).
Conrad et al. (40)	2007	Case–control study	Germany	31 CSCR patients 31 age- and gender-matched control	Symptom Checklist 90-R (SCL-90R)	CSCR patients exhibited higher levels of alexithymia and emotional distress than controls, with alexithymia positively correlated with hostility. Anxiety and depression scores were also significantly higher in the CSCR group (anxiety: *p* = 0.001; depression: p < 0.001).
Garg et al. (42)	2005	Case–control study	India	30 patients with acute CSCR 30 age controls	Zung Self-Rating Depression Scale	Psychological factors were investigated as potential contributors to the etiopathogenesis of CSCR. Depression scores did not differ significantly between CSCR patients and controls (42.46 ± 6.52 vs. 40.92 ± 7.64).
Hufnagel et al. (43)	2024	Case–control study	Germany	65 CSCR patients 19 BRVO patients 19 Ocular Adnexa Disease without Retinal Disease patients	Generalized Anxiety Disorder 7-item scale (GAD-7) Patient Health Questionnaire-9 depression screening (PHQ-9)	When comparing patients with CSCR, BRVO, and ocular adnexal disease without retinal involvement, anxiety and depression scores did not differ significantly among groups at either the 3- or 6-month follow-up. Anxiety was more prevalent in BRVO patients (16%) than in those with CSCR (8%) or healthy controls (5%), whereas depression occurred in 21% of BRVO, 12% of CSCR, and 11% of healthy participants.
Ji et al. (44)	2018	Case–control study	China	134 CSCR patients 134 age-and-sex-matched healthy controls	Depression Anxiety Stress Scales 21-item version (DASS-21)	Anxiety and depression rates were significantly higher in CSCR patients than in controls (anxiety: 28.4% vs. 14.9%, *p* < 0.008; depression: 25.4% vs. 10.4%, p = 0.001). Poor sleep quality, elevated stress levels, and emotional difficulties were also significant among CSCR patients.
Kim et al. (45)	2018	Case–control study	Korea	37 CSCR patients 37 age-and-sex-matched healthy controls	Beck Depression Inventory (BDI) State–Trait Anxiety Inventory (STAI)	Significant differences between active CSCR patients and healthy controls were observed for depression (*p* = 0.037), whereas inactive patients did not differ from controls in psychological functioning. Patients with acute CSCR were also significantly more depressed (*p* = 0.029).
Kuru and Aslan (46)	2021	Case–control study	Turkey	18 CSCR patients 18 Myopic patients 18 age-and-sex-matched healthy controls	Beck Depression Inventory (BDI) Health Anxiety Inventory (HAI) State–Trait Anxiety Inventory (STAI)	Patients with acute CSCR showed higher trait anxiety compared with healthy controls (*p* = 0.048) and myopic patients (*p* = 0.021). No significant differences among the three groups were found for state anxiety (*p* = 0.295), depression (*p* = 0.763), or health anxiety (*p* = 0.405).
Mukherji et al. (47)	2024	Case–control study	India	91 CSCR patients 91 patients with other non-chorioretinal diseases	Hamilton Anxiety Rating Scale (HAM-A) Hamilton Depression Rating Scale (HDRS)	Anxiety was present in 40% of patients, while major depression was observed in 24%. Significant differences between CSCR and non-CSCR groups were found for both anxiety and depression (p < 0.001 for each). Moreover, anxiety and depressive symptoms were significantly associated with lower visual acuity and increased central macular thickness due to subretinal fluid accumulation.
Nongrem et al. (54)	2021	Pilot study (cohort longitudinal design)	India	40 CSCR patients	State–Trait Anxiety Inventory (STAI)	CSCR patients who underwent meditation training showed a reduction in STAI scores (STAI-Y1: *p* = 0.01; STAI-Y2: *p* = 0.008) compared with baseline. However, identical significant differences were also reported for the non-meditation group. Mean and standard deviation values for the non-meditation group remained similar between baseline and follow-up (STAI-Y1: 45.5 ± 1.5 vs. 44.1 ± 3.4; STAI-Y2: 46.5 ± 2.3 vs. 44.2 ± 3.5).
Penas et al. (48)	2022	Case–control study	Portugal	20 CSCR patients 14 age-and-sex-matched controls	State–Trait Anxiety Inventory (STAI)	State and trait anxiety scores were significantly higher in CSCR patients than in controls (STAI-Y1: 38.5 ± 8.4 vs. 25.0 ± 4.8, p = 0.002; STAI-Y2: 44.5 ± 4.9 vs. 32.0 ± 4.6, p = 0.002). Furthermore, a significant negative correlation was found between STAI scores and neurovascular coupling, which was stronger for state anxiety (p = 0.002 vs. *p* = 0.035).
Şahin et al. (49)	2014	Case–control study	Turkey	30 CSCR patients 30 age-gender-matched healthy controls	Symptom Checklist 90-R (SCL 90-R)	Anxiety, phobic anxiety, and depression were significantly higher in patients with CSCR than in controls (anxiety: *p* = 0.003; phobic anxiety: *p* = 0.009; depression: *p* = 0.005). Moreover, CSCR patients reported a lower quality of life compared with healthy controls.
Scarinci et al. (50)	2020a	Case–control study	Italy	14 CSCR patients 14 OSA patients 28 controls	Hamilton Anxiety Rating Scale (HAM-A) Hamilton Depression Rating Scale (HDRS)	CSCR patients showed a significant increase in cortisol production upon awakening. Psychological distress was evident, with patients presenting higher depression scores than controls (*p* < 0.05).
Scarinci et al. (51)	2020b	Case–control study	Italy	17 CSCR patients 17 controls	Beck Depression Inventory-II (BDI-II) Positive and Negative Affect Schedule (PANAS) State–Trait Anxiety Inventory (STAI)	Cortisol Awakening Response (CAR AUCG) values were significantly higher in CSCR patients. Compared with controls, patients showed higher scores in negative affectivity (*p* = 0.023), total depression (BDI-II: *p* = 0.036), and somatic–affective symptoms (BDI-II factor: *p* = 0.043).
Setrouk et al. (52)	2016	Case–control study	France	29 CSCR patients 29 age-gender-matched controls	Hospital Anxiety and Depression Scale (HADS)	No significant differences were found between CSCR patients and controls in anxiety or depression scores.
Yang et al. (53)	2025	Case–control study	United Kingdom	109 CSRR 247 age-matched controls	Hospital Anxiety and Depression Scale (HADS)	No significant differences in anxiety or depression scores were found between CSCR patients and controls (anxiety: 5.37 ± 3.42 vs. 5.52 ± 3.62, *p* = 0.678; depression: 2.76 ± 2.63 vs. 3.05 ± 3.03, *p* = 0.619).

BAI, Beck Anxiety Inventory; BDI, Beck Depression Inventory; BDI-II, Beck Depression Inventory-II; BMI, Body Mass Index; BRVO, Branch Retinal Vein Occlusion; CAR-AUCg, Cortisol Awakening Response; CSCR, Central Serous Chorioretinopathy; DASS-21, Depression Anxiety Stress Scales - 21-item version; GAD-7, Generalized Anxiety Disorder - 7-item scale; HADS, Hospital Anxiety and Depression Scale; HAI, Health Anxiety Inventory; HAM-A, Hamilton Anxiety Rating Scale; HDRS, Hamilton Depression Rating Scale; OSA, Obstructive Sleep Apnea; PANAS, Positive and Negative Affect Schedule; PHQ-9, Patient Health Questionnaire - 9-item depression screening; RD, Diabetic Retinopathy; SCL-90R, Symptom Checklist-90R; STAI, State–Trait Anxiety Inventory; STAI-Y1, State Anxiety subscale; STAI-Y2, Trait Anxiety subscale.

### 3.1 Characteristics of the included studies

Among the included studies, 19 adopted a case–control design (36–53), while one study presented a pilot cohort longitudinal design (54). The selected works were published between 2005 and 2025 and conducted across multiple countries, including China (44), France (52), Germany (40, 41, 43), India (47, 54), Iran (55), Israel (38), Italy (50, 51), Korea (45), Portugal (48), Turkey (36, 37, 39, 46, 49), Switzerland (55), and the United Kingdom (53).

Regarding the assessment of anxiety and depression, a wide range of standardized psychometric tools was employed across studies. The Beck Anxiety Inventory (BAI) was used in three investigations (36–38), whereas the Beck Depression Inventory (BDI) appeared in four (36, 37, 45, 46). One study applied the Beck Depression Inventory-II (BDI-II) (51), and another the Depression Anxiety Stress Scales - 21 item version (DASS-21) (44). The Generalized Anxiety Disorder Scale (GAD-7) was used by Hufnagel et al. (43), while the Hamilton Anxiety Rating Scale (HAM-A) appeared two studies (50, 55), and the Hamilton Depression Rating Scale (HDRS) in two (47, 50). Additional instruments included the Health Anxiety Inventory (HAI) (46), the Hospital Anxiety and Depression Scale (HADS) (52, 53), the Patient Health Questionnaire-9 (PHQ-9) (43), the Positive and Negative Affect Schedule (PANAS) (51), the State–Trait Anxiety Inventory (STAI) (45, 46, 48, 51, 54), the Symptom Checklist-90R (SCL-90R) (39–41, 49), and finally the Zung Self-Rating Depression Scale (42).

Overall, the 20 included studies encompassed a total of 980 patients diagnosed with CSCR, assessed through diverse methodological approaches and psychodiagnostic instruments.

### 3.2 Depression

A total of 17 studies assessed depressive symptoms among patients with CSCR (36, 37, 39–53), all adopting a case–control design.

Most studies reported significantly higher levels of depressive symptoms in CSCR patients compared with control groups. Specifically, 13 studies observed elevated depression scores in individuals with CSCR (36, 37, 39–41, 43–45, 47–51). The majority of these works included matched healthy controls (36, 39–41, 44–46, 48–53), while several studies also compared CSCR patients to other ophthalmologic populations.

In this regard, Hufnagel et al. (43) found that depressive symptoms reached 11% among CSCR patients when compared with individuals affected by BRVO or ocular adnexal disorders without retinal involvement. Mukherji et al. (47) similarly reported higher depression scores in CSCR patients compared to those with non-chorioretinal ophthalmic conditions, and Scarinci et al. (50) observed greater depressive symptomatology in CSCR than in patients with obstructive sleep apnea.

Nevertheless, four studies did not detect statistically significant differences between groups (42, 46, 52, 53), and one study, Balkarli et al. (37), assessed only selected BDI items (including sexual dysfunction), reporting an inverse association with age and BMI rather than group differences.

Taken together, the evidence suggests that depressive symptoms are considerably more prevalent in CSCR patients than in controls in most available studies, indicating that mood alterations may represent a clinically meaningful dimension of this condition and warrant attention for targeted psychological assessment and tailored interventions.

### 3.3 State anxiety

A total of 19 studies investigated state anxiety in patients with CSCR (36–41, 43–55). Eighteen studies adopted a case–control design, comparing CSCR patients with healthy controls or other clinical populations, whereas one study (54) implemented a longitudinal cohort design, exploring the impact of a meditation-based intervention on anxiety levels among CSCR patients exposed or not exposed to the treatment condition.

Among the case–control studies, nine investigations reported significantly higher state anxiety scores in CSCR patients compared to controls (36, 39–41, 44, 47–49, 55) whereas eight others found no significant differences (37, 38, 43, 45, 46, 50–53). In the study by Balkarli et al. (37), anxiety and depression scales were used selectively to assess sexual dysfunction-related items, representing a specific methodological choice rather than a full-scale evaluation of affective variables.

The Nongrem et al. (54) study represents a distinctive case within the literature: it employed the State–Trait Anxiety Inventory (STAI) and involved random assignment of CSCR patients to meditation or control groups. Although its longitudinal design was innovative, some methodological concerns were later raised regarding its statistical robustness and sample size.

Overall, findings on state anxiety were more heterogeneous compared with those on depression. While depressive symptoms tended to show consistent increases across most studies, evidence for elevated anxiety was less uniform, with approximately half of the studies reporting non-significant results. Nevertheless, anxiety remains one of the most prevalent psychological conditions observed in clinical practice and represents a frequent emotional response, vulnerability factor, or comorbid condition in situations of mental distress. Its assessment in CSCR patients is therefore clinically valuable, supporting the need for integrated screening and follow-up interventions aimed at promoting psychological well-being alongside ophthalmological care.

### 3.4 Trait anxiety

Five studies assessed trait anxiety in patients with CSCR. Kim et al. (45) and Scarinci et al. (51) reported no significant differences between CSCR patients and healthy controls. In contrast, Kuru and colleagues (46) found significantly higher levels of trait anxiety in patients with acute CSCR compared to both healthy and myopic control groups. Similarly, Penas et al. (48) confirmed elevated trait anxiety in CSCR and identified a significant negative correlation between trait anxiety and neurovascular coupling, suggesting a possible physiological link between emotional reactivity and vascular regulation. Nongrem et al. (54) also assessed trait anxiety within their longitudinal meditation-based study; however, several methodological limitations were later noted regarding its statistical validity and sample design.

Overall, trait anxiety appears to be a less frequently investigated dimension compared with state anxiety, yet it may hold substantial clinical relevance. Further studies are needed to clarify its specific contribution to disease onset, course, and recovery. Integrating trait anxiety assessment into clinical protocols may enhance the understanding of individual vulnerability profiles and support the development of targeted psychological interventions in patients with CSCR.

## 4 Discussion

Anxiety and depression represent key clinical variables of growing scientific interest, particularly when examined in relation to medical conditions that remain underexplored from a psychological perspective. Their influence on disease adjustment, treatment adherence, and clinical outcomes has been increasingly documented (19, 56–59). Indeed, anxiety and depressive symptoms are well-established risk factors across both chronic and acute medical populations, contributing to worse prognosis, reduced quality of life, and higher recurrence rates (25, 28, 60–63).

In ophthalmology, the psychological burden of visual impairment has been examined in numerous conditions (64–69). However, certain disorders-such as Central Serous Chorioretinopathy (CSCR) still require multifactorial investigation integrating both biological and psychological perspectives. Despite being a relatively common form of non-surgical retinopathy, CSCR has historically received less psychosomatic attention compared with other retinal diseases (66, 70–75).

The present review demonstrated that depressive symptoms were more consistently elevated in CSCR patients than anxiety symptoms. Specifically, most studies investigating depression reported significantly higher scores among CSCR patients compared with controls, with only four studies yielding non-significant results. Even when depression was partially assessed, such as in the study by Balkarli et al. (37), which focused on sexual dysfunction items, the evidence still pointed toward increased depressive vulnerability. These findings align with previous research in other medical conditions, reinforcing the notion that depressive symptomatology is a critical component of psychological adjustment to ocular disease.

When comparing state and trait anxiety, the evidence appeared more heterogeneous. Approximately half of the included studies reported significantly higher anxiety levels in CSCR patients, while the remainder found no differences. The only intervention-based contribution (54), which explored meditation as a stress-reduction technique, provided preliminary but methodologically limited evidence of improvement, as later discussed by Panigrahi (76) and Venkatesh and Surve (77). These mixed results suggest that anxiety responses may fluctuate according to disease phase, perceived controllability, and individual coping mechanisms. Moreover, the personality trait of anxiety sensitivity (related to anxiety but not the same) predicts symptoms of dry eye severity more significantly than anxiety itself and even beyond anxiety (78–80).

The co-occurrence of ophthalmological, anxiety and depression symptoms represents a significant clinical challenge. The findings of this review underscore the need for integrated care models that include psychological screening and evidence-based interventions aimed at mitigating anxiety and depressive symptoms in CSCR patients. Future research should adopt longitudinal and interventional designs to clarify causal relationships and assess the effectiveness of psychotherapeutic or pharmacological treatments tailored to this population.

Several limitations should be acknowledged. Most of the included studies employed a case–control design, limiting the possibility of establishing temporal or causal inferences. Only one study incorporated an interventional framework, and even that did not qualify as a formal psychotherapeutic or psychopharmacological treatment. Furthermore, the statistical approaches were often limited to group comparisons, with few studies applying multivariate or predictive analyses capable of elucidating complex biopsychosocial relationships.

Nevertheless, the evidence synthesized in this review offers a solid foundation for advancing both research and clinical practice. By confirming the consistent association between CSCR and affective symptomatology, these findings highlight the importance of a biopsychosocial perspective in ophthalmology-one that integrates emotional, physiological, and behavioral dimensions to promote holistic patient care.

## 5 Conclusion

This systematic review provides the first comprehensive synthesis of empirical evidence on anxiety and depression in patients with Central Serous Chorioretinopathy (CSCR). Across the analyzed studies, both affective dimensions emerged as clinically relevant, with depression showing a more consistent pattern of elevation than anxiety. Although findings regarding anxiety were more heterogeneous, particularly for state and trait measures, the overall evidence indicates that emotional distress is a frequent and significant correlate of CSCR.

From a clinical standpoint, these results underscore the importance of adopting a biopsychosocial approach to CSCR management. Routine psychological screening for anxiety and depression should be considered in ophthalmological practice to facilitate early detection, improve patient adherence, and promote overall well-being. Integrating psychological support-whether through counseling, stress-reduction programs, or psychotherapeutic interventions-could meaningfully enhance both visual and emotional recovery outcomes.

Despite the consistency of the observed associations, the current literature remains limited in scope and methodological diversity. The predominance of case–control designs restricts causal interpretation, and the scarcity of longitudinal and interventional studies hinders a deeper understanding of the dynamic interplay between psychological and ophthalmological factors. Future research should therefore prioritize prospective, multicentre, and interdisciplinary designs, incorporating standardized diagnostic criteria and validated psychometric tools. Such efforts would not only strengthen the evidence base but also clarify the mechanistic links between stress-related physiological processes, such as cortisol dysregulation and choroidal vascular changes, and affective symptomatology.

In conclusion, the evidence synthesized through this systematic review provides a robust conceptual and empirical foundation for subsequent research and clinical innovation. Recognizing the psychological dimensions of CSCR represents a crucial step toward integrative ophthalmic care, capable of addressing both the biological and emotional needs of patients and fostering a more comprehensive understanding of how mind and vision interact in health and disease.

## Data Availability

The original contributions presented in the study are included in the article/Supplementary material, further inquiries can be directed to the corresponding author.

## References

[1] Frederiksen IN Arnold-Vangsted A Anguita R Boberg-Ans LC Cehofski LJ van Dijk EH . Global incidence of central serous chorioretinopathy: a systematic review, meta-analysis, and forecasting study. Ophthalmol Ther. (2025) 14:2443–67. doi: 10.1007/s40123-025-01220-0 40782298 PMC12413349

[2] Kaye R Chandra S Sheth J Boon CJ Sivaprasad S Lotery A. Central serous chorioretinopathy: an update on risk factors, pathophysiology and imaging modalities. Prog Retin Eye Res. (2020) 79:100865. doi: 10.1016/j.preteyeres.2020.100865, 32407978

[3] Spaide RF Cheung CMG Matsumoto H Kishi S Boon CJ van Dijk EH . Venous overload choroidopathy: a hypothetical framework for central serous chorioretinopathy and allied disorders. Prog Retin Eye Res. (2022) 86:100973. doi: 10.1016/j.preteyeres.2021.100973 34029721

[4] Warrow DJ Hoang QV Freund KB. Pachychoroid pigment epitheliopathy. Retina. (2013) 33:1659–72. doi: 10.1097/IAE.0b013e3182953df4, 23751942

[5] Fung AT Yang Y Kam AW. Central serous chorioretinopathy: a review. Clin Experiment Ophthalmol. (2023) 51:243–70. doi: 10.1111/ceo.14201, 36597282

[6] Khan AH Lotery AJ. Central serous chorioretinopathy: epidemiology, genetics and clinical features. Annu Rev Vis Sci. (2024) 10:907. doi: 10.1146/annurev-vision-102122-102907, 38748934

[7] Samanta A Driban M Sahoo N Parameswarappa D Singh SR Caplash S . Central serous chorioretinopathy and ocular comorbidities. J Clin Med. (2025) 14:720. doi: 10.3390/jcm14030720, 39941390 PMC11818775

[8] Berger L Bühler V Yzer S. Central serous chorioretinopathy–an overview. Klin Monatsbl Augenheilkd. (2021) 238:971–9. doi: 10.1055/a-1531-5605, 34416788

[9] Galletti B Freni F Meduri A Oliverio GW Signorino GA Perroni P . Rhino-orbito-cerebral mucormycosis in diabetic disease mucormycosis in diabetic disease. J Craniofac Surge. (2020) 31:e321–4. doi: 10.1097/SCS.0000000000006191, 32028364

[10] Lange CA Qureshi R Pauleikhoff L. Interventions for central serous chorioretinopathy: a network meta-analysis. Cochrane Database Syst Rev. (2025) 6:11841. doi: 10.1002/14651858.CD011841.pub3 PMC12169103 40522203

[11] Oliverio GW Meduri A De Salvo G Trombetta L Aragona P. OCT angiography features in diabetes mellitus type 1 and 2. Diagnostics. (2022) 12:2942. doi: 10.3390/diagnostics12122942, 36552948 PMC9777069

[12] Zhang X Lim CZF Chhablani J Wong YM. Central serous chorioretinopathy: updates in the pathogenesis, diagnosis and therapeutic strategies. Eye Vision. (2023) 10:33. doi: 10.1186/s40662-023-00349-y, 37430344 PMC10334588

[13] Brinks J van Dijk EH Kiełbasa SM Mei H van der Veen I Peters HA . The cortisol response of male and female choroidal endothelial cells: implications for central serous chorioretinopathy. J Clin Endocrinol Metab. (2022) 107:512–24. doi: 10.1210/clinem/dgab670 34546342 PMC8764349

[14] Kimura T Araki T Komuku Y Iwami H Gomi F. Central serous chorioretinopathy and blood serotonin concentrations. J Clin Med. (2021) 10:558. doi: 10.3390/jcm10040558, 33546112 PMC7913142

[15] Jain M Mohan S van Dijk EH. Central serous chorioretinopathy: pathophysiology, systemic associations, and a novel etiological classification. Taiwan J Ophthalmol. (2022) 12:381–93. doi: 10.4103/2211-5056.362601, 36660127 PMC9843580

[16] Sesar A Sesar AP Jurisic D Cvitkovic K Cavar I. Unraveling the puzzle of central serous chorioretinopathy: exploring psychological factors and pathophysiological mechanisms. Med Sci Monit. (2023) 29:e941216–1. doi: 10.12659/MSM.941216, 37515320 PMC10395186

[17] Bower JE Kuhlman KR. Psychoneuroimmunology: an introduction to immune-to-brain communication and its implications for clinical psychology. Annu Rev Clin Psychol. (2023) 19:331–59. doi: 10.1146/annurev-clinpsy-080621-045153, 36791765

[18] Di Giuseppe M Spatola C Merlo EM Silvestro O Giorgianni CM Juli G . Research advances in clinical psychology of chronic diseases. Psychiatr Danub. (2025) 37:56–62. 40982873

[19] Helgeson VS Zajdel M. Adjusting to chronic health conditions. Annu Rev Psychol. (2017) 68:545–71. doi: 10.1146/annurev-psych-010416-044014 28051935

[20] Caputo A Vicario CM Cazzato V Martino G. Psychological factors as determinants of medical conditions, volume II. Front Psychol. (2022) 13:865235. doi: 10.3389/fpsyg.2022.865235, 35386893 PMC8977586

[21] Cohen S Herbert TB. Health psychology: psychological factors and physical disease from the perspective of human psychoneuroimmunology. Annu Rev Psychol. (1996) 47:113–42. doi: 10.1146/annurev.psych.47.1.113, 8624135

[22] Conversano C Di Giuseppe M. Psychological factors as determinants of chronic conditions: clinical and psychodynamic advances. Front Psychol. (2021) 12:635708. doi: 10.3389/fpsyg.2021.635708, 33584488 PMC7876054

[23] Merlo EM Myles LA Martino G. On the critical nature of psychosomatics in clinical practice. Clin Psychol Europe. (2025) 7:1–3. doi: 10.32872/cpe.16309, 36405677

[24] Stanton AL Revenson TA Tennen H. Health psychology: psychological adjustment to chronic disease. Annu Rev Psychol. (2007) 58:565–92. doi: 10.1146/annurev.psych.58.110405.085615, 16930096

[25] Gold SM Köhler-Forsberg O Moss-Morris R Mehnert A Miranda JJ Bullinger M . Comorbid depression in medical diseases. Nat Rev Dis Primers. (2020) 6:69. doi: 10.1038/s41572-020-0200-2, 32820163

[26] Merlo EM Myles LAM Silvestro O Ruggeri D Russo GT Squadrito G . Type 1 diabetes mellitus and alexithymia: a systematic review. Healthcare. (2025) 13:2402. doi: 10.3390/healthcare13192402, 41095488 PMC12523671

[27] Pedro Costa A da Silva Brito I Mestre TD Matos Pires A José Lopes M. Meshing anxiety, depression, quality of life, and functionality in chronic disease. Healthcare. (2025) 13:539. doi: 10.3390/healthcare13050539, 40077101 PMC11898782

[28] Scott AJ Correa AB Bisby MA Dear BF. Depression and anxiety trajectories in chronic disease: a systematic review and meta-analysis. Psychother Psychosom. (2023) 92:227–42. doi: 10.1159/000533263, 37607505

[29] Silvestro O Vicario CM Costa L Sparacino G Lund-Jacobsen T Spatola CA . Defense mechanisms and inflammatory bowel diseases: a narrative review. Res Psychother. (2025) 28:854. doi: 10.4081/ripppo.2025.854, 40178111 PMC12203894

[30] Silvestro O Lund-Jacobsen T Ferraù F Blanca ES Catalano A Sparacino G . Anxiety, depression and acromegaly: a systematic review. J Endocrinol Investig. (2025) 48:527–46. doi: 10.1007/s40618-024-02483-3, 39509066

[31] Genovese G Meduri A Muscatello MRA Gangemi S Cedro C Bruno A . Central serous chorioretinopathy and personality characteristics: a systematic review of scientific evidence over the last 10 years (2010 to 2020). Medicina. (2021) 57:628. doi: 10.3390/medicina57060628, 34208694 PMC8235071

[32] Pandolfo G Genovese G Bruno A Palumbo D Poli U Gangemi S . Sharing the same perspective. Mental disorders and central serous chorioretinopathy: a systematic review of evidence from 2010 to 2020. Biomedicine. (2021) 9:1067. doi: 10.3390/biomedicines9081067, 34440271 PMC8394052

[33] Liberati A Altman DG Tetzlaff J Mulrow C Gøtzsche PC Ioannidis JP . The PRISMA statement for reporting systematic reviews and meta-analyses of studies that evaluate healthcare interventions: explanation and elaboration. BMJ. (2009) 339:b2700. doi: 10.1136/bmj.b2700 19622552 PMC2714672

[34] Moher D Liberati A Tetzlaff J Altman DG. Preferred reporting items for systematic reviews and meta-analyses: the PRISMA statement. BMJ. (2009) 339:e1000097. doi: 10.1136/bmj.b2535, 21603045 PMC3090117

[35] Page MJ McKenzie JE Bossuyt PM Boutron I Hoffmann TC Mulrow CD . The PRISMA 2020 statement: an updated guideline for reporting systematic reviews. BMJ. (2021) 372:n71. doi: 10.1136/bmj.n71 33782057 PMC8005924

[36] Balkarli A Erol MK Yucel O Akar Y. Frequency of fibromyalgia syndrome in patients with central serous chorioretinopathy. Arq Bras Oftalmol. (2017) 80:4–8. doi: 10.5935/0004-2749.20170003, 28380092

[37] Balkarli A Erol MK Yalcinkaya S Erol RS. Frequency of erectile dysfunction in males with central serous chorioretinopathy. Semin Ophthalmol. (2018) 33:482–7. doi: 10.1080/08820538.2017.1301968, 28328282

[38] Ben-Eli H Asher T Lender R Mirsky D Abu-Shkara R Hamuda M . Anterior segment characteristics and quality of life of patients with central serous Chorioretinopathy. J Clin Med. (2025) 14:1812. doi: 10.3390/jcm14061812, 40142620 PMC11943245

[39] Çam F Sevik M Aykut A Dericioğlu V Çam CŞ Şahin Ö. Dysfunctional personality beliefs and psychopathology in patients with central serous chorioretinopathy. J Fr Ophtalmol. (2024) 47:103997. doi: 10.1016/j.jfo.2023.05.032, 37919151

[40] Conrad R Weber NF Lehnert M Holz FG Liedtke R Eter N. Alexithymia and emotional distress in patients with central serous chorioretinopathy. Psychosomatics. (2007) 48:489–95. doi: 10.1176/appi.psy.48.6.489, 18071095

[41] Conrad R Geiser F Kleiman A Zur B Karpawitz-Godt A. Temperament and character personality profile and illness-related stress in central serous chorioretinopathy. Sci World J. (2014) 2014:631687. doi: 10.1155/2014/631687, 24696654 PMC3947818

[42] Garg S Dada T Talward D Nainiwal S Tewari HK Dube S. Psychological factors in the etiopathogenesis of central serous chorioretinopathy. Ann Ophthalmol. (2005) 37:201–5. doi: 10.1385/AO:37:3:201

[43] Hufnagel HJ Lahmann C Agostini H Lange C Pauleikhoff LJ group RnCs. Psychometric assessment of patients with central serous chorioretinopathy and correlation with disease stage and progression: a case control study. BMC Ophthalmol. (2024) 24:92. doi: 10.1186/s12886-024-03356-2, 38424605 PMC10902987

[44] Ji Y Li M Zhang X Peng Y Wen F. Poor sleep quality is the risk factor for central serous chorioretinopathy. J Ophthalmol. (2018) 2018:9450297. doi: 10.1155/2018/9450297, 30155284 PMC6093041

[45] Kim Y-K Woo SJ Park KH Chi YK Han JW Kim KW. Association of central serous chorioretinopathy with psychosocial factors is dependent on its phase and subtype. Korean J Ophthalmol. (2018) 32:281–9. doi: 10.3341/kjo.2017.0144, 30091306 PMC6085183

[46] Kuru T Aslan F. Acute central serous Chorioretinopathy and psychological parameters: chicken and egg dilemma. J Retina Vitreous. (2021) 30:55–60. doi: 10.37845/ret.vit.2021.30.9

[47] Mukherji S Karmakar S Dasgupta S. Association of central serous chorioretinopathy with type of personality, anxiety and depression. Indian J Ophthalmol. (2024) 72:S60–5. doi: 10.4103/IJO.IJO_1180_23, 38131544 PMC10833166

[48] Penas SC Resende JA Sousa AR Carneiro ÂV Reis FF. Central serous chorioretinopathy and angioid streaks: coincidental? BMC Ophthalmol. (2022) 22:359. doi: 10.1186/s12886-022-02566-w, 36064394 PMC9442979

[49] Şahin A Bez Y Kaya MC Türkcü FM Şahin M Yüksel H. Psychological distress and poor quality of life in patients with central serous chorioretinopathy. Semin Ophthalmol. (2014) 29:73–6. doi: 10.3109/08820538.2013.793728, 23758338

[50] Scarinci F Patacchioli FR Ghiciuc CM Pasquali V Bercea RM Cozma S . Psychological profile and distinct salivary cortisol awake response (CAR) in two different study populations with obstructive sleep apnea (OSA) and central serous Chorioretinopathy (CSC). J Clin Med. (2020) 9:2490. doi: 10.3390/jcm9082490, 32756367 PMC7464438

[51] Scarinci F Patacchioli FR Palmery M Pasquali V Costanzo E Ghiciuc CM . Diurnal trajectories of salivary cortisol and α-amylase and psychological profiles in patients with central serous chorioretinopathy. Chronobiol Int. (2020) 37:510–9. doi: 10.1080/07420528.2019.1702553, 31842621

[52] Setrouk E Hubault B Vankemmel F Zambrowski O Nazeyrollas P Delemer B . Circadian disturbance and idiopathic central serous chorioretinopathy. Graefes Arch Clin Exp Ophthalmol. (2016) 254:2175–81. doi: 10.1007/s00417-016-3378-y, 27207466

[53] Yang Y Foster VS Marlowe S Stevenson SR Alexander I Foster RG . Sleep and mood in central serous chorioretinopathy. Eye. (2025) 39:1615–23. doi: 10.1038/s41433-025-03688-3, 40011741 PMC12089360

[54] Nongrem G Surve A Venkatesh P Sagar R Yadav RK Chawla R . Effect of short-term meditation training in central serous chorioretinopathy. Indian J Ophthalmol. (2021) 69:3559–63. doi: 10.4103/ijo.IJO_3499_20, 34826995 PMC8837379

[55] Bazzazi N Ahmadpanah M Akbarzadeh S Seif Rabiei MA Holsboer-Trachsler E Brand S. In patients suffering from idiopathic central serous chorioretinopathy, anxiety scores are higher than in healthy controls, but do not vary according to sex or repeated central serous chorioretinopathy. Neuropsychiatr Dis Treat. (2015) 11:1131–6. doi: 10.2147/NDT.S83216, 25995637 PMC4425338

[56] Carroll S Moon Z Hudson J Hulme K Moss-Morris R. An evidence-based theory of psychological adjustment to long-term physical health conditions: applications in clinical practice. Psychosom Med. (2022) 84:547–59. doi: 10.1097/PSY.0000000000001076, 35412516

[57] Dekker J de Groot V. Psychological adjustment to chronic disease and rehabilitation–an exploration. Disabil Rehabil. (2018) 40:116–20. doi: 10.1080/09638288.2016.1247469, 27830936

[58] Livneh H. Psychosocial adaptation to chronic illness and disability: an updated and expanded conceptual framework. Rehabil Counsel Bull. (2022) 65:171–84. doi: 10.1177/00343552211034819

[59] Santana L Fontenelle LF. A review of studies concerning treatment adherence of patients with anxiety disorders. Patient Prefer Adherence. (2011) 5:427–39. doi: 10.2147/PPA.S23439, 21949606 PMC3176182

[60] Cobham VE Hickling A Kimball H Thomas HJ Scott JG Middeldorp CM. Systematic review: anxiety in children and adolescents with chronic medical conditions. J Am Acad Child Adolesc Psychiatry. (2020) 59:595–618. doi: 10.1016/j.jaac.2019.10.010, 31676391

[61] Hughes M Brown SL Campbell S Dandy S Cherry MG. Self-compassion and anxiety and depression in chronic physical illness populations: a systematic review. Mindfulness. (2021) 12:1597–610. doi: 10.1007/s12671-021-01602-y

[62] Lotfaliany M Bowe SJ Kowal P Orellana L Berk M Mohebbi M. Depression and chronic diseases: co-occurrence and communality of risk factors. J Affect Disord. (2018) 241:461–8. doi: 10.1016/j.jad.2018.08.011, 30149333

[63] Shahar G. Interdisciplinarity and integration: an introduction to the special issue on psychopathology in medical settings. J Clin Psychol Med Settings. (2021) 28:1–5. doi: 10.1007/s10880-020-09752-2, 33219478 PMC7678582

[64] Basilious A Xu CY Malvankar-Mehta MS. Dry eye disease and psychiatric disorders: a systematic review and meta-analysis. Eur J Ophthalmol. (2022) 32:1872–89. doi: 10.1177/11206721211060963, 34935549 PMC9297048

[65] Constable PA Al-Dasooqi D Bruce R Prem-Senthil M. A review of ocular complications associated with medications used for anxiety, depression, and stress. Clin Optom. (2022) 14:13–25. doi: 10.2147/OPTO.S355091, 35237084 PMC8884704

[66] Ramesh PV Morya AK Azad A Pannerselvam P Devadas AK Gopalakrishnan ST . Navigating the intersection of psychiatry and ophthalmology: a comprehensive review of depression and anxiety management in glaucoma patients. World J Psychiatry. (2024) 14:362–9. doi: 10.5498/wjp.v14.i3.362, 38617979 PMC11008383

[67] Shah N Tran E Aly M Phu V Laughlin E Malvankar-Mehta MS. Depression and anxiety in patients with irreversible vision loss: meta-analysis and systematic review. Int J Psychiatry Med. (2025) 2025:82653. doi: 10.1177/00912174251382653 PMC13400842 41061694

[68] Tang WSW Lau NXM Krishnan MN Chin YC Ho CSH. Depression and eye disease—a narrative review of common underlying pathophysiological mechanisms and their potential applications. J Clin Med. (2024) 13:3081. doi: 10.3390/jcm13113081, 38892791 PMC11172702

[69] Tran E Maher A Basilious A Malvankar-Mehta MS. Prevalence of anxiety and depression symptoms in age-related macular degeneration patients: a systematic review and meta-analysis. Arch Ment Health. (2025) 26:14–28. doi: 10.4103/amh.amh_80_24

[70] Groff ML Choi B Lin T Mcllraith I Hutnik C Malvankar-Mehta MS. Anxiety, depression, and sleep-related outcomes of glaucoma patients: systematic review and meta-analysis. Can J Ophthalmol. (2023) 58:346–55. doi: 10.1016/j.jcjo.2022.02.010, 35305959

[71] Li S Liu H Zhu X. The effect of psychotherapy on anxiety, depression, and quality of life in patients with diabetic retinopathy: a protocol for systematic review and network meta-analysis. Medicine. (2021) 100:e28386. doi: 10.1097/MD.0000000000028386, 34941170 PMC8702293

[72] Senra H Macedo AF Nunes N Balaskas K Aslam T Costa E. Psychological and psychosocial interventions for depression and anxiety in patients with age-related macular degeneration: a systematic review. Am J Geriatr Psychiatry. (2019) 27:755–73. doi: 10.1016/j.jagp.2019.03.001, 31005495

[73] Wan K Chen L Young A. Depression and anxiety in dry eye disease: a systematic review and meta-analysis. Eye. (2016) 30:1558–67. doi: 10.1038/eye.2016.186, 27518547 PMC5177754

[74] Zheng Y Wu X Lin X Lin H. The prevalence of depression and depressive symptoms among eye disease patients: a systematic review and meta-analysis. Sci Rep. (2017) 7:46453. doi: 10.1038/srep46453, 28401923 PMC5388862

[75] Zigiotti GL Cavarretta S Morara M Nam SM Ranno S Pichi F . Standard enucleation with aluminium oxide implant (bioceramic) covered with patient’s sclera. Sci World J. (2012) 2012:481584. doi: 10.1100/2012/481584 PMC3361278 22654614

[76] Panigrahi PK. Comment on: effect of short-term meditation training in central serous chorioretinopathy. Indian J Ophthalmol. (2022) 70:1856. doi: 10.4103/ijo.IJO_3016_21, 35502102 PMC9332941

[77] Venkatesh P Surve A. Response to comment on: effect of short-term meditation training in central serous chorioretinopathy. Indian J Ophthalmol. (2022) 70:1857. doi: 10.4103/ijo.IJO_269_22, 35502103 PMC9332994

[78] Reiss S Bootzin RR. Theoretical issues in behavior therapy In: Reiss S Bootzin RR, editors. Theoretical issues in behavior therapy. New York: Academic Press (1985). 107–21.

[79] Toth M Jokić-Begić N Krašić S. The relationship between anxiety sensitivity, emotional states, and dry eye disease symptom severity: a cross-sectional study. Vision. (2025) 9:36. doi: 10.3390/vision9020036, 40265404 PMC12015891

[80] Toth M Jokić-Begić N. Psychological contribution to understanding the nature of dry eye disease: a cross-sectional study of anxiety sensitivity and dry eyes. Health Psychol Behav Med. (2020) 8:202–19. doi: 10.1080/21642850.2020.1770093, 34040868 PMC8114394

[81] De Luca L Mancini M Pandolfo G Genovese G Melita F Carlà MM . Personality Traits, Psychological Stress, and Anatomical Biomarkers in Central Serous Chorioretinopathy: A Multimodal Case–Control Study. Mediterranean Journal of Clinical Psychology. (2025) 13. doi: 10.13129/2282-1619/mjcp-5120, 40142620

